# Application and Future Utilization of Shellac in Orthodontics: A Systematic Review

**DOI:** 10.3390/jcm13102917

**Published:** 2024-05-15

**Authors:** Martin Baxmann, Zoltán Baráth, Krisztina Kárpáti

**Affiliations:** 1 Department of Orthodontics, Faculty of Education and Research, DTMD University, 9516 Wiltz, Luxembourg; 2 Department of Prosthodontics, Faculty of Dentistry, University of Szeged, 6720 Szeged, Hungary; barath.zoltan@stoma.szote.u-szeged.hu; 3 Department of Orthodontics and Pediatric Dentistry, Faculty of Dentistry, University of Szeged, 6720 Szeged, Hungary; karpati.krisztina@stoma.szote.u-szeged.hu

**Keywords:** 3D printing in dentistry, biodegradable materials, eco-friendly orthodontics, orthodontics, shellac, sustainable dental materials

## Abstract

**Background:** This review examines the application of shellac in orthodontics, focusing on its properties, advantages, and potential as an alternative to conventional materials. In orthodontics, where bond strength, ease of application, and removal are paramount, shellac’s capabilities meet these needs while supporting environmentally friendly practices. **Methods:** With objectives centered on evaluating shellac’s effectiveness, biocompatibility, and impact on patient outcomes, a comprehensive search across multiple databases was conducted, including PubMed, Scopus, and Web of Science. This study’s selection criteria targeted studies assessing shellac’s use in orthodontic applications, measuring treatment effectiveness, biocompatibility, and patient satisfaction while excluding those not directly involving orthodontic applications or lacking empirical data. **Results:** Through a qualitative synthesis of the extracted data—encompassing study design, sample size, treatment outcomes, and adverse effects—the findings reveal shellac’s potential benefits in orthodontics, such as enhanced patient comfort and comparable treatment outcomes to traditional materials. However, the review also notes variability in study designs and outcomes, indicating the need for further research. **Conclusions:** This study concluded that shellac presents a promising alternative in orthodontic materials, recommending additional studies to standardize assessment methodologies and confirm its long-term advantages.

## 1. Introduction

The evolution of techniques, new technologies, and materials used in orthodontics reflects a continuous quest to optimize treatment efficacy, patient comfort, and biocompatibility. Shellac, a natural resin secreted by the female lac bug on trees in the forests of India and Thailand, exemplifies this progression [1]. Historically, shellac found early dental applications in the 19th century, particularly within prosthodontics for denture bases, prized for its ease of manipulation and acceptable aesthetics [2]. Although less documented, shellac’s role in orthodontics is equally significant. Before modern adhesives, orthodontic practices sought materials that provided reliable bonding strength, ease of use, and comfort for the patient [3]. Shellac, with its natural origin and presumed biocompatibility, was a favored choice, although its use in this area was sporadically documented [4].

Recent advancements have repositioned shellac within the material sciences domain, highlighting its potential as a light-cured resin suitable for 3D printing applications due to its curing range of 385–405 nm [5]. Shellac’s inherent properties—natural adhesive qualities, biodegradability, and non-toxicity—align with current demands for sustainable and biocompatible materials in medical applications [6]. In orthodontics, where bond strength, ease of application, and removal are paramount, shellac’s capabilities meet these needs while supporting environmentally friendly practices. The hypoallergenic nature of shellac further addresses concerns about allergic reactions sometimes associated with synthetic orthodontic adhesives [7]. As material science in orthodontics evolves to emphasize patient-centered and eco-friendly approaches, the resurgence of traditional materials like shellac is timely, especially given its novel applications in 3D printing [5]. This systematic review aims to consolidate and critically evaluate the literature on shellac in orthodontics, exploring its historical applications and potential for modern demands by answering the following PICO question: “In patients undergoing orthodontic treatment (P), how does the use of shellac (I), compared to conventional orthodontic materials (C), affect the effectiveness, biocompatibility, and patient outcomes (O)?”.

## 2. Materials and Methods

This review systematically encompassed studies that specifically investigated the use of shellac in orthodontic applications. Eligible studies included experimental and observational studies, case reports, clinical trials, and reviews. Given the universal application of orthodontic procedures, the participant criterion was not limited to any specific demographic. Interventions considered involved using shellac in any form within the orthodontic treatment and comparing it with or without alternative materials. The outcomes assessed included, but were not limited to, efficacy, biocompatibility, patient outcomes, and adhesive properties.

This systematic review was performed in accordance to the PRISMA guidelines. Specific inclusion and exclusion criteria were applied to ensure the relevancy and quality of the literature reviewed. Inclusion criteria were set to identify studies that directly assessed the effectiveness, biocompatibility, or environmental impact of shellac used in orthodontic applications and were published in English to ensure accessibility and standard comprehension. To capture recent advancements, this study included experimental, observational studies, case reports, clinical trials, and systematic reviews published within the last 20 years. Exclusion criteria involved omitting studies that did not focus specifically on shellac in orthodontics, such as those exploring other dental uses without comparing shellac, articles without empirical data like opinion pieces, inaccessible full-texts, and duplicate studies to maintain data integrity and scientific rigor.

A comprehensive search was conducted using databases known for their extensive medical and dental literature coverage. These included PubMed, Scopus, Web of Science, and the Cochrane Library. Grey literature sources and conference proceedings were also searched to ensure a thorough exploration, and a hand search of key orthodontic journals was undertaken. Search terms included “Shellac”, “Orthodontics”, “Dental Applications”, and “Adhesives”. Specific search phrases were employed, such as “Shellac in orthodontic treatments”, “Use of shellac in dental braces”, and “Biocompatibility of shellac in orthodontic procedures”. Boolean operators were used to refine the search: for instance, (“Shellac” OR “natural resin”) AND (“orthodontics” OR “dental braces”) AND (“adhesive properties” OR “clinical applications”) NOT “paint”.

Study selection followed a two-stage screening process involving one screener. Initially, titles and abstracts were reviewed for relevance. Subsequently, full-text articles were reviewed to assess compliance with the eligibility criteria. Data from selected studies were extracted using a standardized form. Extracted data included study characteristics (authors, year of publication), methodology, participant demographics, details of the intervention (type, duration, dosage of shellac used), comparison groups, outcomes measured, and results. The quality of included studies was assessed using appropriate tools: the Cochrane Risk of Bias tool for randomized trials, the Newcastle–Ottawa Scale for observational studies, and the RoBDEMAT tool [8,9,10]. Each study was evaluated for potential selection, performance, detection, attrition, and reporting biases.

## 3. Results

**Figure 1:**
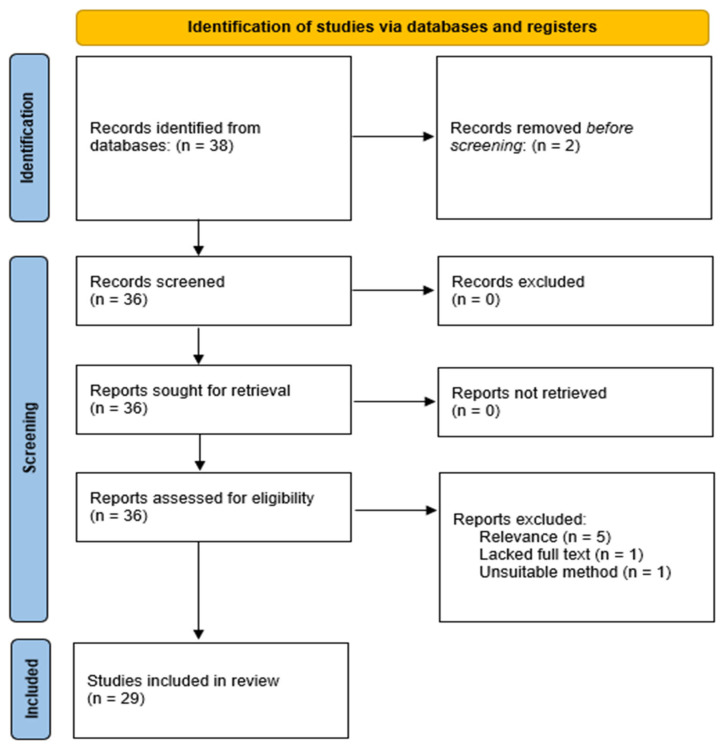
PRISMA flow diagram.

In this systematic review, 38 studies were initially identified, of which 2 were excluded due to duplication. The remaining 36 underwent rigorous screening against the inclusion criteria, excluding 7 articles for reasons such as lack of relevance to the specific application of shellac in orthodontics, absence of full-text availability, or incompatible study design. Consequently, 29 articles were included for comprehensive synthesis (Figure 1). These encompassed a variety of study designs, including cross-sectional, experimental, observational, one safety data sheet (Ettlingen, DE), and systematic reviews (Table 1). The investigations primarily assessed shellac’s biocompatibility, mechanical properties, and role as a sustainable material in orthodontics.

## 4. Discussion

### 4.1. Early Application and Developments

The historical journey of shellac in orthodontics is an intriguing tale of innovation and material evolution. Originating from the secretion of the lac bug, shellac was primarily utilized in the dental field during the late 19th and early 20th centuries [37]. Its initial application was predominantly in prosthodontics, valued for its ease of manipulation and satisfactory aesthetic qualities [12]. In orthodontics, shellac’s role was notably marked by its use in manufacturing dental impressions and retainers [30]. Its natural adhesive properties were first explored in the early 1900s, offering a rudimentary yet effective solution for bracket attachment [19]. Azouka et al. highlight how shellac’s ease of use and removal made it a preferred choice in an era when orthodontic practices were still evolving [2].

The use of shellac in orthodontics also mirrored broader trends in dental material science. As noted by Marin (2023), the preference for natural materials in the early days of dentistry was largely due to their biocompatibility and lower cost [26]. Shellac, being a natural resin, was well-aligned with these preferences.

The transition from shellac to more contemporary materials in orthodontics reflects the technological advancements and growing scientific understanding of material properties. The mid-20th century ushered in a new era of synthetic materials, driven by the need for improved durability, strength, and patient comfort [26]. The advent of acrylic resins and, later, the development of composite materials marked a significant shift in orthodontic practices [37]. These materials offered enhanced bonding strength, reduced treatment times, and greater ease of manipulation than shellac [26].

Moreover, the focus on patient-specific treatments and the rising demand for aesthetic orthodontics fueled the search for materials that were not only effective but also visually appealing [36]. This demand gradually phased out the use of shellac, as it could not compete with newer materials’ color stability and customizability [36]. However, recent trends toward sustainable and biocompatible materials have sparked a renewed interest in shellac [21]. Researchers like Thombare et al. argue that with advancements in material processing and treatment techniques, there is potential for the reintegration of shellac into certain orthodontic applications, particularly in scenarios where biodegradability and minimal environmental impact are prioritized [37].

### 4.2. Physical and Chemical Properties of Shellac

Shellac, a complex natural bioadhesive, presents an intriguing composition primarily comprising resin, wax, and coloring matter. Its resin component, predominantly responsible for its adhesive properties, consists of a complex mixture of hydroxy aliphatic and sesquiterpene acids [37]. Researchers like Obradovic et al. have identified over 35 different components in shellac, with the major constituent being the pleuritic acid, which contributes significantly to its adhesive characteristics [28].

The unique molecular structure of shellac, characterized by long-chain hydrocarbons, imparts several desirable properties. According to Thombare et al., these include a natural semi-transparency, flexibility, and a relatively low melting point, making them easy to manipulate in various orthodontic applications [37]. As Farag and Leopold highlighted, shellac’s thermal properties indicate its softening at around 75 °C and complete melting at about 120 °C, which aligns well with orthodontic procedures requiring material molding [20].

A noteworthy addition to shellac’s properties is its versatility in color variations. This feature is particularly advantageous for coloring 3D printing resins or serving as a colored coating for non-colored print objects, enhancing both aesthetic appeal and functional utility in various applications [5,18]. The natural coloring matter in shellac, though varying based on the processing, generally offers an aesthetic advantage in orthodontic applications, as discussed by Thombare et al. [37].

Furthermore, the wax component of shellac adds to its non-toxic nature. Obradovic et al. noted that the wax contributes to its hypoallergenic profile, making it a potential candidate for patients’ sensitivities to synthetic materials [28]. The potential of shellac as an antibacterial coating also deserves mention. This aspect could significantly impact its application in orthodontics, addressing concerns regarding bacterial growth and hygiene in oral environments.

The adhesive properties of shellac have been a focal point in its application in orthodontics. Chiba et al. describe shellac’s adhesion mechanism as primarily mechanical, adhering well to porous surfaces and providing a satisfactory bond strength for light orthodontic forces [16]. The findings by Lee et al. corroborate this, reporting the bond strength of shellac as adequate for temporary orthodontic applications but not comparable to modern synthetic adhesives for long-term use [23].

An interesting characteristic of shellac’s adhesive quality is its solubility in alcohol and certain other organic solvents, which offers ease of application and removal [20,37]. However, this also poses a limitation in the oral environment, where moisture can compromise its adhesive strength. As Luangtana et al. point out, this necessitates the exploration of shellac derivatives or formulations that are more resistant to moisture for effective orthodontic use [24].

The biodegradability of shellac, a significant factor in its environmental footprint, also impacts its adhesive properties. According to Thombare et al., the biodegradable nature of shellac is advantageous from an ecological perspective [37]. However, it raises concerns about its long-term stability and reliability in orthodontic treatments, especially in moisture-rich oral environments.

In exploring the biomechanical properties of various orthodontic materials, it is crucial to compare the efficacy of traditional materials like shellac with modern alternatives. The study by Xia et al. provides a finite element analysis comparing clear aligners and fixed appliances for anterior retraction, highlighting significant differences in tooth displacement and torque control [38]. This comparative framework is useful in evaluating shellac’s potential in orthodontic treatments. For instance, while shellac has been primarily noted for its biocompatibility and ease of use, understanding its mechanical properties in light of such studies could lead to enhanced applications that balance traditional benefits with modern mechanical requirements. By considering these findings, we can better understand where shellac might fit within the spectrum of orthodontic materials, particularly in cases requiring detailed control of tooth movement and alignment stability.

### 4.3. Clinical Applications of Shellac in Orthodontics

The use of shellac in bonding orthodontic brackets has been a subject of interest in the historical context of orthodontic treatments. A notable example of such innovation is highlighted in a recent case report by Pothuri Sr et al., which discusses the management of Class II Division 2 subdivision malocclusions using an asymmetrical extraction protocol. This approach underscores the ongoing development of orthodontic treatment strategies and provides a relevant contrast to traditional methods, where materials like shellac were used [39]. Examining these contemporary treatment methodologies alongside traditional uses of shellac can offer valuable insights into the potential integration of new and old techniques in orthodontics, especially in handling complex cases where tailored approaches are required.

While modern adhesives predominantly consist of synthetic materials like polymers, shellac was once a go-to material due to its natural adhesive properties [13]. Its initial application in bracket bonding was appreciated for its ease of use and removal. It was particularly beneficial during the early stages of orthodontic treatment when temporary bonding was required [11].

However, studies have shown that while shellac provides sufficient adhesive strength for light orthodontic forces, it may not be ideal for long-term bonding required in contemporary orthodontic treatments [31]. The bond strength of shellac, as observed by several studies, often fell short compared to that of modern synthetic adhesives, especially under the challenging conditions of the oral environment, such as moisture and varying pH levels [20,24,37]. Furthermore, research by Syed et al. highlighted the potential for allergic reactions with synthetic adhesives, suggesting a niche for shellac in patients with specific sensitivities [35]. This biocompatibility aspect of shellac could be leveraged in cases where patient-specific material considerations are crucial.

In addition to these historical uses, shellac has been identified as a type IIb medical product. Base plate shellac is classified as a Class I medical device, indicating its suitability for long-term intraoral use. This categorization opens new avenues for shellac’s application in modern orthodontics, particularly in 3D printing. With its potential for coating 3D-printed orthodontic appliances, shellac could play a significant role in enhancing the quality of these appliances [5]. Reducing surface roughness may help minimize bacterial adhesion, thus improving oral hygiene and reducing the risk of dental caries and gingival diseases [22].

Shellac application has been explored in retention appliances, albeit to a lesser extent than bracket bonding. The flexibility and ease of molding of shellac made it a candidate for use in temporary retainers and space maintainers [30]. Despite these applications, the contemporary use of shellac in retention appliances is limited. The evolution of dental materials has introduced more durable and less reactive materials better suited to the long-term and consistent use that retention appliances require [4].

The biocompatibility of shellac in orthodontic applications, particularly concerning allergic reactions and sensitivities, is a crucial aspect of its clinical viability. Shellac, a natural resin, is generally considered to have a lower propensity to induce allergic reactions than some synthetic orthodontic materials [29]. However, even natural materials can trigger sensitivities in certain individuals, albeit at a significantly lower frequency than synthetics. A study by Mercader-García et al. has shown that allergic reactions to shellac are rare but can occur, particularly in individuals with a history of allergies to similar organic compounds [27]. These reactions range from mild localized irritation to more severe forms like allergic contact dermatitis [27]. The specific component in shellac responsible for such reactions is often difficult to isolate, given its complex chemical composition. Moreover, the risk of sensitization over time, especially with prolonged exposure, is an area that requires more research. Long-term studies are needed to fully understand the allergenic potential of shellac when used in orthodontic applications.

### 4.4. Efficacy and Effectiveness

The efficacy of shellac in orthodontics, particularly regarding its bond strength and durability, is a crucial metric for its clinical utility. While shellac exhibits adequate initial bonding strength for light orthodontic applications, its long-term durability under the mechanical stresses of orthodontic forces is debatable [16]. Also, shellac’s tensile and shear strengths are generally lower than modern synthetic adhesives [16]. This limitation makes shellac less suitable for applications requiring robust long-term bonding, such as fixed orthodontic appliances. Moreover, the durability of shellac is also impacted by its biodegradation properties. The natural degradation process of shellac in the oral environment can lead to a reduction in adhesive strength over time, necessitating more frequent adjustments or replacements of the orthodontic appliance [32].

Recent advancements and ongoing research in the field of biomaterials, however, have opened new possibilities for the application of shellac in orthodontics. There is growing interest in exploring the potential of shellac in enhancing the biomechanical properties of orthodontic appliances. Innovations in this area focus on modifying the shellac formulation or combining it with other materials to improve its mechanical properties and strength [14]. By doing so, it might be possible to retain shellac’s biocompatibility and natural advantages while overcoming its traditional limitations.

Researchers are investigating ways to utilize shellac to create more comfortable and effective orthodontic appliances. This includes the development of shellac-based composites or coatings that could offer improved performance in terms of flexibility, strength, and patient comfort. Such modifications could lead to a new generation of orthodontic materials that blend the natural benefits of shellac with the enhanced functionality required for modern orthodontic practices.

Moreover, aesthetics in orthodontic appliances cannot be overstated, particularly for pediatric patients. Colorful and visually appealing devices have been shown to significantly enhance patient compliance and preference, as evidenced in studies focusing on pediatric orthodontics. For instance, research by Schott and Menne on patient preferences in orthodontic appliances underscores the impact of aesthetic factors on treatment adherence, especially among younger patients [33].

In this context, shellac’s ability to be produced in various colors presents a unique advantage. The versatility in color options allows for a level of customization that resonates with the aesthetic preferences of diverse patient groups. This is particularly crucial for children, who are more likely to comply with orthodontic treatment when given a choice in how their appliance looks visually. Lyros et al. highlight this aspect with their study, suggesting that color and design can improve adherence to treatment protocols among young patients [25].

Furthermore, integrating shellac’s color properties with modern technology, such as 3D printing, opens new avenues in orthodontic treatment. The ability to customize colors enhances the appeal of 3D-printed orthodontic devices, marrying functionality with aesthetic appeal [5,18]. This synergy between shellac’s aesthetic properties and advanced manufacturing technologies allows for efficiently producing patient-specific appliances. The result is a functional orthodontic device that is aesthetically pleasing and more likely to be accepted and worn consistently by patients, particularly children.

These considerations show that shellac’s varied coloration capabilities and integration with modern manufacturing methods like 3D printing can significantly improve patient compliance, especially in pediatric orthodontics. This aspect of shellac, therefore, represents an important factor in the ongoing evolution of orthodontic materials and treatment approaches. As this field evolves, it is anticipated that shellac, either in its pure form or as part of composite materials, will play a significant role in the future of orthodontic treatments.

### 4.5. Sustainability and Environmental Impact

The sustainability and environmental impact of orthodontic materials have become increasingly important in the context of global environmental concerns. As a natural, biodegradable substance, shellac has a significantly lower environmental footprint, as determined by various other industry applications [15,34]. The production and disposal of shellac-based materials involve minimal environmental hazards, aligning with the growing demand for eco-friendly medical and dental materials [34]. Shellac’s biodegradability confers a substantial environmental advantage, particularly in reducing plastic waste commonly associated with orthodontic treatments [17]. Therefore, if the use of shellac leads to more frequent appliance replacements or adjustments, the resultant environmental benefits may be mitigated.

In sustainable orthodontic practices, the role of shellac is being re-envisioned, especially in the context of 3D printing. The recent emphasis on sustainability in dentistry and orthodontics includes exploring traditional materials like shellac and employing them innovatively. Applying shellac in 3D printing orthodontic appliances is a prime example of this approach [5]. By utilizing shellac in 3D-printed appliances, it is possible to harness its environmental benefits while leveraging advanced manufacturing techniques. This addresses the environmental impact of orthodontic treatments and opens up new avenues for applying shellac in custom, patient-specific appliances.

Moreover, the integration of shellac in 3D printing underscores a broader theme in contemporary orthodontic practices: blending traditional, eco-friendly materials with cutting-edge technology [5]. This approach aligns with the growing interest in sustainable healthcare solutions and the shift towards more environmentally conscious medical practices. As the field of orthodontics continues to evolve, using shellac in such innovative applications could contribute significantly to reducing the ecological footprint of orthodontic treatments and promoting sustainable practices in the dental industry.

To appropriately incorporate the study “Accuracy and Completeness of ChatGPT-Generated Information on Interceptive Orthodontics: A Multicenter Collaborative Study”, you should reference it in the section of your article that discusses future trends, the role of emerging technologies in orthodontics or specifically, the integration of artificial intelligence (AI) in orthodontic practice and education. This study can offer a significant perspective on how AI tools like ChatGPT could supplement traditional orthodontic training and patient education, highlighting both the potential and limitations of AI in clinical settings.

### 4.6. Limitations and Future Directions

The research on the use of shellac in orthodontics faces certain limitations. A major issue is the scarcity of long-term clinical studies. Much of the knowledge about shellac in orthodontics is based on short-term studies, historical data, and applications in other industries. More comprehensive, long-term clinical trials are needed to fully understand the implications of using shellac, particularly in terms of its long-term efficacy and safety. The lack of comparative studies that pit shellac against a wide range of modern orthodontic materials also provides another limitation. Such comparative research is essential to accurately position shellac within the available materials, assessing its strengths and weaknesses in various clinical scenarios.

Furthermore, shellac is a natural resin with variations in its composition depending on the source and processing methods. This variability can lead to inconsistencies in research findings, making it challenging to draw generalized conclusions about its use in orthodontics. Overcoming these challenges requires innovative approaches in material science and application techniques and a concerted effort to conduct methodologically robust, long-term clinical research. In conclusion, the future of shellac in orthodontics lies in its innovation and exploration of new applications. With advancements in material science and a better understanding of its properties, shellac could see a resurgence in orthodontic applications, offering a blend of traditional and modern approaches to meet the evolving needs of orthodontic care.

Also, the RoBDEMAT tool assesses the risk of bias, focusing on four domains: planning and allocation, specimen preparation, outcome assessment, and data treatment and outcome reporting. This assessment helped to identify some areas of potential bias, thus contributing to the review’s overall analysis. The evaluation indicated that while planning and allocation in the included studies generally attempted randomization and blinding, there were occasional lapses that might introduce some degree of selection and allocation biases. Specimen preparation was largely consistent with established protocols, although minor deviations were noted, potentially introducing performance bias. Outcome assessment was evaluated for consistency in reporting and measuring the effectiveness and biocompatibility of shellac. While most studies adhered to a standard methodology, some inconsistencies were observed, which could affect the measurement accuracy. Finally, the analysis of data treatment and outcome reporting showed a mostly transparent approach, but some instances of incomplete reporting were identified, raising concerns about possible reporting biases. The use of the RoBDEMAT tool highlighted areas where the robustness of the studies could be questioned, indicating that while the body of evidence had strengths, it also had certain limitations that could affect the conclusions drawn from the review.

Additionally, as orthodontics continues to evolve with technological advancements, the integration of artificial intelligence (AI) has shown the potential to revolutionize several aspects of clinical practice and education. A pertinent study assessing AI’s capability in the field is by Hatia et al., which evaluates the accuracy and completeness of ChatGPT-generated responses to complex questions in interceptive orthodontics. This multicenter collaborative study involving ten specialized orthodontists from various Italian universities demonstrated that while AI could provide high levels of accuracy (median score of 4.9/6) and completeness (median score of 2.5/3) in solving clinical scenarios, it also highlighted the technology’s current limitations in replacing human judgment and expertise. The results suggest that although AI, like ChatGPT, exhibits substantial promise in enhancing educational tools and supporting clinical decision-making, its utility is supplementary, emphasizing the necessity for skilled human oversight [40]. By integrating AI tools responsibly, orthodontic professionals can leverage these technologies to enhance treatment planning and patient education and improve treatment outcomes while maintaining a critical oversight role.

## 5. Conclusions

This systematic review comprehensively synthesizes shellac’s historical and contemporary applications in orthodontics, affirming its biocompatibility, environmental benefits, and viability as a biodegradable material. This study’s analysis of 29 articles, spanning a variety of research designs including cross-sectional, experimental, and observational, underscores shellac’s potential to enhance patient comfort and achieve comparable treatment outcomes to traditional materials.

The results indicate that shellac, with its inherent properties such as natural adhesive qualities and non-toxicity, aligns well with the current demands for sustainable and biocompatible materials in medical applications. For example, the studies reviewed highlighted how shellac’s hypoallergenic nature potentially mitigates allergic reactions commonly associated with synthetic orthodontic adhesives, providing a safer alternative for sensitive patients.

Moreover, the innovative use of shellac in 3D printing technology exemplifies the integration of traditional materials with modern techniques and opens new avenues for creating custom. These patient-specific orthodontic appliances are environmentally sustainable. This integration is particularly promising in the context of our findings, where shellac-based 3D printed models demonstrated improved biomechanical properties and enhanced patient-specific treatment options.

The evolving landscape of material science in orthodontics, which now emphasizes patient-centered approaches and eco-friendly materials, supports a renewed interest in shellac. This review establishes a solid foundation for future research and application, suggesting that with further development and standardization of assessment methodologies, shellac could significantly contribute to clinical excellence and environmental stewardship in orthodontics.

Thus, the current study not only reaffirms shellac’s relevance in modern orthodontic applications but also sets the stage for its expanded role in future orthodontic solutions. It encourages ongoing dialogue and collaborative research efforts to explore shellac’s full potential, ensuring that the orthodontic field progresses in a scientifically sound and ecologically responsible manner.

## Figures and Tables

**Table 1:** Articles included in the review.

Author(s)	Year of Publication	Methodology	Intervention/Observation	Results
Aravindakshan et al. [11]	2021	Experimental	Bonding strength of shellac	Shellac concentration provided the maximum bonding strength of 12.5 MPa
Arbree et al. [12]	1996	Cross-sectional	Current participant techniques in complete denture prosthodontics regarding preliminary and final impressions, record bases, and denture teeth	Seventy-four percent of the respondents used only irreversible hydrocolloid (alginate) for their preliminary impressions; fifteen percent used only modeling plastic impression compound. Eighty-one percent used only modeling plastic impression compound for border molding of the final impression tray; seven percent used only polyether impression material. Forty-eight percent used only polysulfide rubber (PR) impression material for their final impression material; four percent used only polyether impression material. Only 1 school still used shellac as one of its materials for record bases. Thirty-five percent used only Triad, thirty-five percent used only acrylic resin, and twenty-four percent used both materials.
Ardelean et al. [13]	2014	Review	Alternatives to classic resin	Thermoplastic materials such as polyamides (nylon), acetal resins, epoxy resins, polystyrene, polycarbonate resins, polyurethane, and acrylic thermoplastic resins were introduced in dentistry as an alternative to classic resins, which have major disadvantages such as the toxicity of the residual monomer, awkward wrapping system, and difficult processing.
Azouka et al. [2]	1993	Review	A historical review of the general uses of shellac is presented, as well as the various manufacturing processes, properties, and chemical composition.	Shellac’s ease of use and removal made it a preferred choice in an era when orthodontic practices were still evolving.
Bar and Bianco-Peled [14]	2020	Experimental	Test a solution to improve the biomechanical properties of shellac.	Jeffamine, an agent that facilitates hydrophilic plasticizing and amine interaction with shellac carboxyl groups, improved solubility, mechanical properties, and thermal stability over 18 months.
Bar and Bianco-Peled [15]	2021	Observational	Investigate the physicochemical characteristics of shellac.	Shellac is composed of a mix of polyesters and single esters of hydroxy-aliphatic and sesquiterpenoid acids. Over time, its mechanical and physicochemical properties break down, causing it to become brittle and decrease in solubility.
Chansatidkosol et al. [5]	2022	Experimental	Assess the feasibility of applying shellac as a biopolymer filament for fused deposition modeling (FDM) 3D printing.	The results demonstrated that shellac with an initial heat of 80 degrees Celsius for 15 min and annealed at 80 degrees Celsius for 12 h had similar properties. However, the annealed sample at 80 degrees Celsius for 24 h had a lower acid value and formed an insoluble solid. The findings of this study suggest that shellac would make suitable FDM filaments.
Chiba et al. [16]	2003	Experimental	Adhesive strength of shellac to tooth surfaces	The adhesive strength on untreated enamel was 5 MPa regardless of the shellac concentration; etching treatment increased the strength to 13.7 MPa; the optimal concentration of shellac with the maximum adhesive strength is about 0.1 to 1%.
Cho et al. [17]	2023	Experimental	Miscibility properties of polyacrylonitrile blending films with biodegradable polymer, shellac	Blending with other polymer materials increases shellac’s biodegradability.
DETAX GmbH and Co. [18]	2015	Safety data sheet	Safety data for Luxaprint shellac color	Shellac is available in various colors and is suitable for medical devices or orthodontic appliances.
Doubleday [19]	1998	Review	Impression materials used by the orthodontic profession; assessments of alginates, silicones, and bite registration materials	Shellac initially offered a rudimentary yet effective solution for bracket attachment.
Farag and Leopold [20]	2007	Observational	Physiochemical properties of various shellac types	Shellac raw material is provided in its acid form, which is subject to an unpredictable change in the chemical structure
Irimia-Vladu et al. [21]	2013	Review	The use of the natural resin shellac	Natural resin shellac is an organic substrate that demonstrates biocompatibility.
Khawwam et al. [22]	2023	Experimental	Comparison of polished orthodontic appliance surfaces and the number of biofilms present with different cleaning solutions.	The cleaning solution used was the same. The study found that polished versus unpolished appliances reduced bacterial biofilm formation.
Lee et al. [23]	1991	Experimental	The difference in tensile bond strength between the composite resin veneer and the cast Ni-Cr disk when different bead adhesives were used to make the laboratory patterns	No significant difference in the mean tensile bond strength was observed between the Visio-Gem and shellac groups; the higher tensile bond strength in the cyanoacrylate group is thought to be attributed to the low rheological property of the adhesive that allowed greater exposure of the bead for retention; using different adhesives in the fabrication of composite resin veneered-castings may affect the bond strength in the composite resin-metal interface.
Luangtana et al. [24]	2007	Experimental	Increase the stability of shellac	The applications of salt forms proved statistically significant in reducing the polymerization process, whereas certain plasticizers could enhance the stability.
Lyros et al. [25]	2023	Review	Present and comment on various means of retention in orthodontics, discussing different types of retainers and their efficacy	While different types of retainers (like Hawley, vacuum-formed, and fixed retainers) have specific applications and benefits, no conclusive evidence suggests one type of retainer is universally more effective than others. It emphasizes the need for individualized retainer selection based on patient-specific factors and underscores the importance of patient cooperation and oral hygiene in successful orthodontic retention.
Marin [26]	2023	Review	The historical evolution of dental biomaterials, as well as to understand the reasons behind their biocompatibility and identify the key factors that have influenced their development and use over the past 5000 years	This review covers more than 30 centuries of technological advances in oral biomaterials, from Egyptian gold-wire appliances to advanced cobalt alloys and polymer formulations still in use today.
Mercader-García et al. [27]	2023	Cross-sectional	Shellac allergies	Shellac appears to be a prevalent allergen in patients with suspected contact dermatitis related to cosmetics or foodstuff, as 20% of the sample had a positive allergic reaction to shellac.
Obradovic et al. [28]	2017	Experimental	The composite efficiency of two shellac-based models	A high content of cellulose and low concentrations of ethanol and polyethylene glycol produced biocomposites with high-stress resistance and a high Young’s modulus, whereas a low content of cellulose and a high concentration of additives gave samples with a low Young’s modulus and high elasticity.
Olms [29]	2019	Observational	To determine the frequencies and symptoms of allergies to dental materials	The most common allergies were to metals, of which nickel and cobalt were the most common allergens. Furthermore, many allergies were indicated to ingredients of cosmetics and composites.
Paradowska-Stolarz et al. [30]	2022	Review	Overview of the recent advances in the field of natural polymers used to maintain or restore oral health	The highly desirable properties of natural polymers, such as availability, the capability of chemical modifications, biodegradability, and biocompatibility, make them very attractive materials. They could be used in almost every field of dentistry, including caries management, periodontology, prosthodontics, and the regeneration and reconstruction of oral tissues.
Rahardjo et al. [31]	2022	Experimental	The use of biocomposite (Bovine Hydroxyapatite (BHA)/Agave Cantula fibre/shellac) as a dental material	The biocomposite can be used as a dental material after further development.
Selvaraj et al. [32]	2023	Review	Corrosion of orthodontic appliances in the oral environment	A nonspecific aging pattern, including the calcification of absorbed ion complexes and proteinaceous debris, is anticipated when orthodontic materials are exposed to the oral cavity; this could influence the morphologic, structural, and compositional traits, including the mechanical properties of orthodontic alloys and polymers.
Schott and Menne [33]	2018	Cross-sectional	Investigate how patient-selected colors for removable appliances affect wear times and behavior, particularly in a pediatric population.	Color choice did not significantly influence the overall wear times of the appliances. While patients freely selected colors, wear times varied widely. The study revealed no significant age or gender-related patterns in wear behavior or compliance related to the selected color.
Skaf et al. [34]	2023	Review	Explore the use of shellac as a sustainable, greener material	Shellac is an advantageous material that is green and sustainable.
Syed et al. [35]	2015	Review	Develop a systematic approach for selecting and monitoring dental materials available in the market, thereby giving insight into predicting their risk of inducing allergic reactions.	Amalgam is the dental material reported to cause the most adverse reactions in patients, and the incidence of oral lichenoid reactions adjacent to amalgam restorations occurs more often than with other dental materials.
Thomas [36]	2015	Review	The artistic part of the orthodontic science	A focus on patient-specific treatments and the rising demand for aesthetic orthodontics fuelled the search for materials that were effective and visually appealing, leading to the investigation of shellac in the field.
Thombare et al. [37]	2022	Review	Properties, applications, and future potential for shellac	Being natural, non-toxic, eco-friendly, tasteless, odorless, and versatile, shellac has tremendous potential to be used in several industries.

## Data Availability

No new data were created.
